# Investigation of the effect of the structure of large-area carbon nanotube/fuel composites on energy generation from thermopower waves

**DOI:** 10.1186/1556-276X-9-536

**Published:** 2014-09-30

**Authors:** Hayoung Hwang, Taehan Yeo, Jo-Eun Um, Kang Yeol Lee, Hong-Seok Kim, Jae-Hee Han, Woo-Jae Kim, Wonjoon Choi

**Affiliations:** 1 School of Mechanical Engineering, Korea University, 145 Anam-ro, Seongbuk-gu, Seoul 136-713, Republic of Korea; 2 Department of Chemical and Biological Engineering, Gachon University, 1342 Seongnamdaero, Sujeong-Gu, Seongnam, Gyeonggi-do 461-701, Republic of Korea; 3 Department of Energy IT, Gachon University, 1342 Seongnamdaero, Sujeong-Gu, Seongnam, Gyeonggi-do 461–701, Republic of Korea

**Keywords:** Carbon nanotube, Alignment of nanostructures, Thermopower waves, Combustion, Energy conversion

## Abstract

Thermopower waves are a recently developed energy conversion concept utilizing dynamic temperature and chemical potential gradients to harvest electrical energy while the combustion wave propagates along the hybrid layers of nanomaterials and chemical fuels. The intrinsic properties of the core nanomaterials and chemical fuels in the hybrid composites can broadly affect the energy generation, as well as the combustion process, of thermopower waves. So far, most research has focused on the application of new core nanomaterials to enhance energy generation. In this study, we demonstrate that the alignment of core nanomaterials can significantly influence a number of aspects of the thermopower waves, while the nanomaterials involved are identical carbon nanotubes (CNTs). Diversely structured, large-area CNT/fuel composites of one-dimensional aligned CNT arrays (1D CNT arrays), randomly oriented CNT films (2D CNT films), and randomly aggregated bulk CNT clusters (3D CNT clusters) were fabricated to evaluate the energy generation, as well as the propagation of the thermal wave, from thermopower waves. The more the core nanostructures were aligned, the less inversion of temperature gradients and the less cross-propagation of multiple thermopower waves occurred. These characteristics of the aligned structures prevented the cancellation of charge carrier movements among the core nanomaterials and produced the relative enhancement of the energy generation and the specific power with a single-polarity voltage signal. Understanding this effect of structure on energy generation from thermopower waves can help in the design of optimized hybrid composites of nanomaterials and fuels, especially designs based on the internal alignment of the materials. More generally, we believe that this work provides clues to the process of chemical to thermal to electrical energy conversion inside/outside hybrid nanostructured materials.

## Background

Chemical combustion technologies deliver a high energy density, and the output can be directly converted into a number of types of mechanical and thermal energy. When combustion is integrated with moving parts, electrical energy can be produced. Most research at the micro/nanoscale has focused on the conversion of chemical energy into mechanical or thermal energy by combustion because the mechanical apparatus required for the chemical-to-electrical energy conversion are difficult to be integrated at the micro/nanoscale. There have been serious efforts to utilize combustion as the main energy source for various micro/nanoscopic applications, such as microthrusters [1,2], microreactors [3], and microactuators [4]. Further, nanostructured materials or devices have been widely explored as additives for the amplification of combustion [5] or to enhance the reaction rate during combustion [6,7]. Recently, a new micro/nanoscopic energy conversion concept, thermopower waves, has been proposed, in which the combustion of the chemical fuel in specially designed nanostructures can produce concomitant electrical energy without the need for any mechanical parts [8,9]. The dynamic temperature and chemical potential gradient established by thermal wave propagation [10] accelerates charge carriers through the nanostructured materials, which results in electrical energy generation. It was reported that the peak output voltage and specific power per mass associated with this approach are very high, and thermopower waves have the potential to be applied in the miniaturized power sources required for specific micro/nanosystems [9]. Therefore, research on thermopower waves has focused on the enhanced energy generation obtainable by applying high-Seebeck-coefficient materials in the room-temperature to high-temperature regime.

For high-Seebeck-coefficient materials in the low-temperature regime, Bi_2_Te_3_ films and Bi_2_Te_3_-Sb_2_Te_3_ have been used as the core thermoelectric materials for generating thermopower waves, and the maximum output voltages reached 150 and 200 mV, respectively [11]. The serial connection of core-shell structures of multi-walled carbon nanotubes (MWCNTs) and Sb_2_Te_3_ increased the peak output voltage [12] by up to 400 mV. As for the high-Seebeck-coefficient materials in the high-temperature regime, metal oxides such as ZnO [13,14] and MnO_2_[15] have been employed to enhance the output voltage. The output voltage of ZnO was as large as 500 mV, while a MnO_2_ film prepared using MnO_2_ powders provided 1.8 V. Although the core thermoelectric materials play an important role in the generation of thermopower waves, other factors which implement the thermopower waves, such as the core material alignment and the chemical fuel composition, can also affect the fundamental phenomena and response.

In this work, we explored the effects that different arrangements of nanostructures, composed of networks of CNTs, have on the different aspects of the energy generation, temperature gradient, and reaction velocity, as well as mutual correlation in thermopower waves. A one-dimensional aligned CNT array (1D CNT array), a randomly oriented CNT film (2D CNT film), and a randomly aggregated bulk CNT cluster (3D CNT cluster) were fabricated to be the core materials of the thermopower wave devices, and layers of chemical fuel were deposited on the CNT surfaces using a wet impregnation process. During the combustion of the chemical fuel, the temperature gradient at the beginning position and at the ending position in the 1D CNT array and 2D CNT film was 500°C to 700°C, and preheating along the CNTs increased the temperatures of the CNTs prior to the reaction. Due to cooling behind the reacted region and the thermal wave propagation, the temperature gradient was inverted in thermopower waves; this resulted in an inverted output voltage signal, which was normally observed in the 2D CNT film and 3D CNT cluster. The output voltages and specific powers of the 1D CNT arrays showed the highest values among the three types of CNT-based nanostructures due to the effective energy transfer in the alignment direction without any inversion of the temperature gradient. Moreover, the energy generation from thermopower waves depends on the structural characteristics of the CNT network, regardless of the total mass of the CNT/fuel composites. Our experimental investigation of the effects of the structure of large-area CNT/fuel composites revealed that the alignment and arrangement of nanostructured networks significantly changes the output voltage, peak specific power, and temperature distribution as well as the reaction propagation velocity. These results suggest that miniaturized sources of thermopower waves have the capability to maintain or enhance the energy generation, because the overall performance does not depend on the total mass of the specific system. Thus, one of these sources can be used as a scalable power source. This work indicates specific strategies for obtaining the ideal alignment of nanostructured materials for the optimized generation of thermopower waves.

## Methods

**Figure 1:**
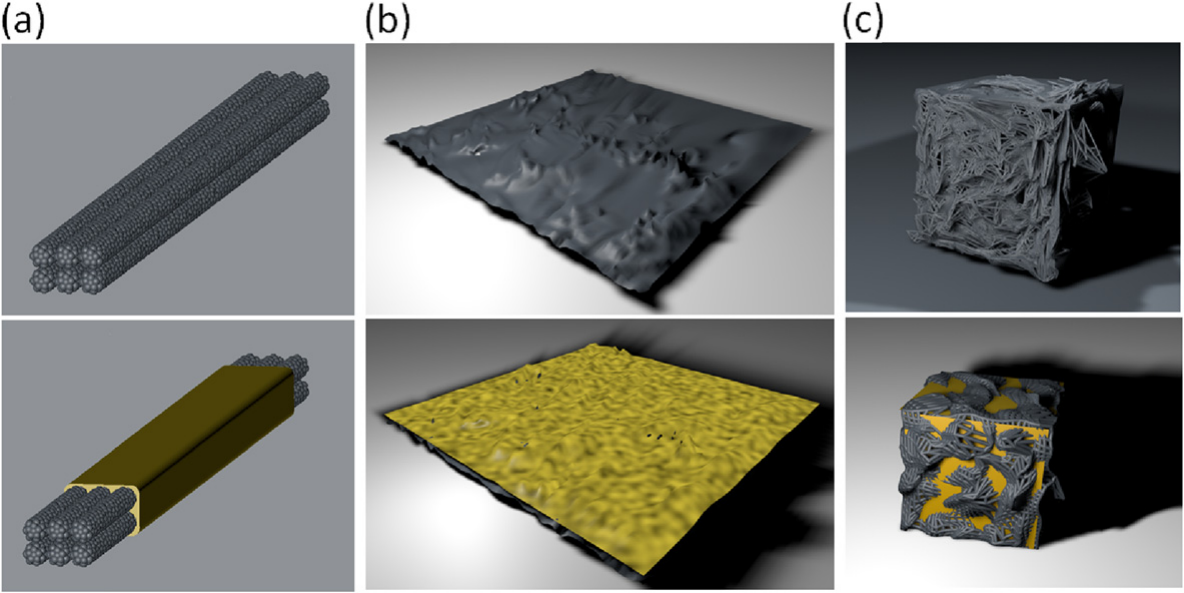
**Schematic diagrams of the 1D, 2D, and 3D CNT/fuel composites. (a)** One-dimensional aligned CNTs (top) and fuel-clad composite (bottom), **(b)** two-dimensional randomly oriented CNT film (top) and CNT/fuel film composite (bottom), and **(c)** three-dimensional randomly aggregated bulk CNT cluster (top) and CNT/fuel cluster composite (bottom).

To investigate the effect of the structure of the core nanomaterials, three different types of CNT networks: a 1D CNT array, a 2D CNT film, and a 3D CNT cluster, were designed and evaluated as the core materials for thermopower wave generation. Schematic representations of these three types of CNT networks and the corresponding hybrid composites with the chemical fuels are shown in Figure 1. The wet impregnation of the structures with the fuel, dissolved in solution, was conducted by the permeation of the chemical fuel through the CNT networks. To confirm the independence of the structural effect with respect to the chemical fuel, two chemical fuel mixtures, picric acid/sodium azide and nitrocellulose/sodium azide, were applied to the 1D, 2D, and 3D CNT networks (Figure 1a,b,c).

### Preparation of a 1D CNT array

**Figure 2:**
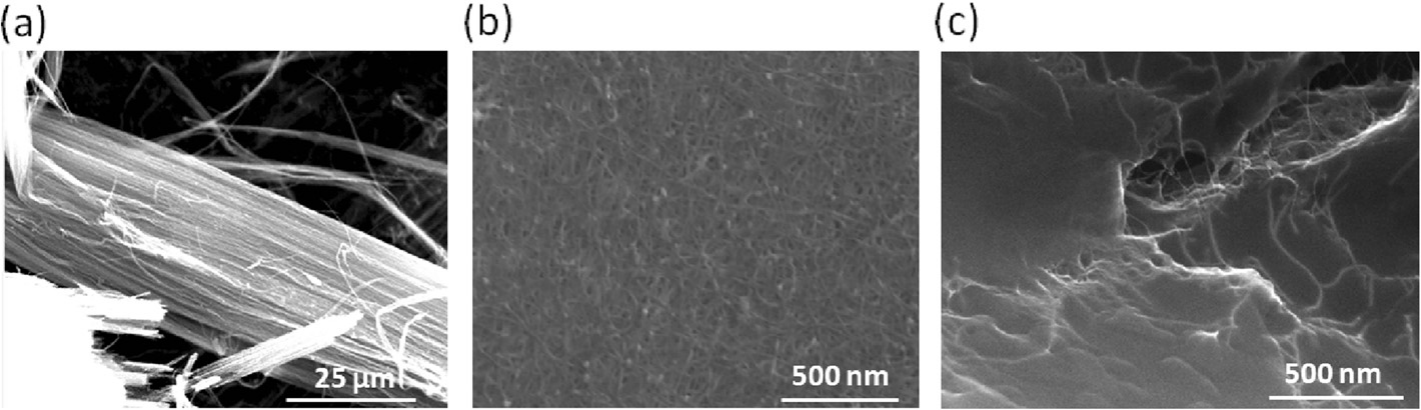
**SEM images of the different types of fabricated CNT networks. (a)** One-dimensional aligned CNTs, **(b)** two-dimensional randomly oriented CNT film, and **(c)** three-dimensional randomly aggregated bulk CNT cluster.

Vertically aligned CNTs (VACNTs) were synthesized by the thermal chemical vapor deposition (TCVD) method [16]. The deposition of 10-nm-thick Al_2_O_3_ and 1-nm-thick Fe layers on a silicon wafer was conducted by e-beam evaporation. Ethylene was the carbon source, and Joule heating by tungsten at the entrance of the quartz tube promoted the decomposition of the carbon. Argon was used as the carrier gas, and hydrogen formed the nanoparticles on the Fe layer, the roots of the MWCNTs. After the TCVD process, a freestanding VACNT array was obtained by separating the CNT layer from the silicon wafer. The length of the VACNT array was 3 to 5 mm, while the cross-sectional area of the 1D CNTs was controlled by the post-processing treatment physically, without any chemical treatment. Figure 2a shows an electron micrograph of the 1D CNT array.

### Preparation of 2D CNT films

CNTs were dispersed in deionized (DI) water with surfactants (2 wt.% sodium dodecyl sulfate or sodium cholate) by homogenization for 1 h at 6,500 rpm. After a cuphorn sonicator was used to disperse the CNTs for 10 min, density gradient ultracentrifugation was applied to remove the CNT bundles [17]. The decanted solution was used as the base solution to fabricate a randomly oriented CNT film by vacuum filtration. The decanted CNT solution was filtered through a 25-mm anodisc membrane (Anodisc, Whatman, Piscataway, NJ, USA), and repetitive washing with DI water removed the surfactants remaining inside the CNT film. The as-prepared CNT film on the alumina membrane was floated on a 3-M NaOH solution that dissolved the entire alumina membrane. After complete removal of the alumina membrane, the NaOH solution was replaced with DI water using repetitive circulation. The floating CNT film was transferred to a silicon wafer underneath the CNT film by draining the DI water in the bath [18]. An electron micrograph of the percolated network of CNTs in this 2D CNT film is shown in Figure 2b.

### Preparation of 3D CNT clusters

Randomly aggregated bulk CNT clusters were fabricated, starting with the above method used for CNT purification [19,20]. Nitric acid was applied to the dispersed CNTs, which floated on the top of the solution. This mixture was homogenized with a vortex mixer, and the CNTs agglomerated in a single spongelike ball. After DI water cleaning, the aggregated CNTs were formed into a rectangular shape by drying the ball in air for 1 day. An electron micrograph of a 3D CNT cluster thus obtained is shown in Figure 2c.

### Preparation of CNT/fuel composites

**Figure 3:**
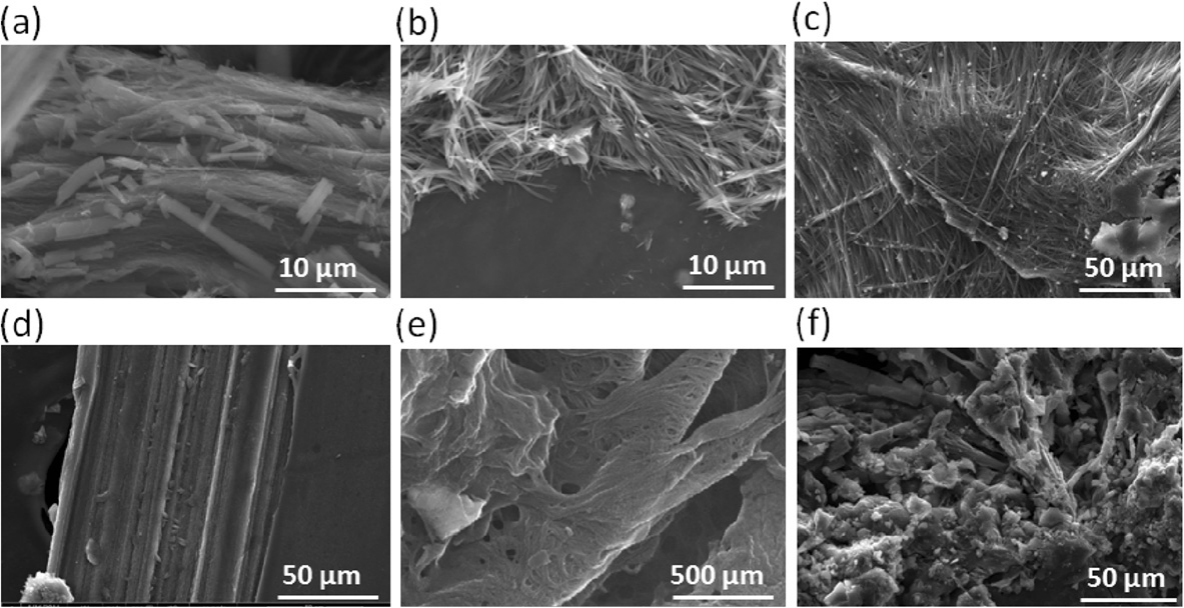
**SEM images of the CNT/fuel composites. (a)** 1D, **(b)** 2D, and **(c)** 3D CNT/picric acid/sodium azide composites and **(d)** 1D, **(e)** 2D, and **(f)** 3D CNT/nitrocellulose/sodium azide composites.

Two different combinations of combustible fuels were used to fabricate the CNT/fuel composites. The first mixture was picric acid and sodium azide. Picric acid was selected as the main chemical fuel due to its large enthalpy of combustion (2,570 kJ/mol) [21], and sodium azide was selected as the primary igniter due to its low activation energy (40 kJ/mol). Repetitive wet impregnation of these chemicals in solution (2.6 mM of picric acid and 0.3 mM of sodium azide dissolved in an acetonitrile solution and DI water, respectively) saturated the chemical fuels throughout the CNT networks and formed the densely packed CNT structures via strong van der Waals and capillary forces among the CNTs. After the complete evaporation of solvents, the 1D, 2D, and 3D CNT/fuel composites, incorporating the same chemical fuel (picric acid/sodium azide), were obtained, as shown in Figure 3a,b,c. The second fuel mixture was nitrocellulose and sodium azide. The impregnation method was identical to that applied to fabricate CNT/fuel composites above, and the results are shown in Figure 3d,e,f. The nitrocellulose (dissolved in acetonitrile) induced more aggregation and stacking because it had a higher viscosity than the picric acid solution. However, in both cases, the fuel mixtures permeated among the CNT network and formed the CNT/fuel composites.

### Thermopower wave measurements

The thermopower wave measurement was conducted using three main pieces of equipment: an oscilloscope (Tektronix DPO2004B, Tektronix Inc., Beaverton, OR, USA, to measure the real-time voltage generation), a high-speed CCD camera system (Nikon Phantom V7.3-8GB color camera, macrolens 105 mm, f/2.8D, Nikon Corp., Tokyo, Japan, to observe the overall aspects of the combustion), and two optical pyrometers (Raytek MM1MHCF1L and MM2MLCF1L, Raytek Corp., Santa Cruz, CA, USA, to record the real-time change in temperature of the CNT structures) at specific positions during thermopower wave propagation. The reaction front of the thermal waves passed the first and second optical pyrometers, in turn, at the ignition position and the ending position of the propagation, respectively. It was assumed that the time at which the temperature reached its maximum value indicated the time of the passing of the reaction front during combustion. The as-prepared 1D, 2D, and 3D CNT/fuel composites were fixed on silicon wafers, and copper films were used as electrodes. Silver paste was used as an adhesive to join the ends of the CNT structures and the copper electrodes. Combustion was initiated by tungsten Joule heating at the leading edge of the CNT/fuel structures. Because the tungsten element only contacted the chemical fuel layer, and not the CNTs, there was no electrical disturbance from Joule heating.

## Results and discussion

**Figure 4:**
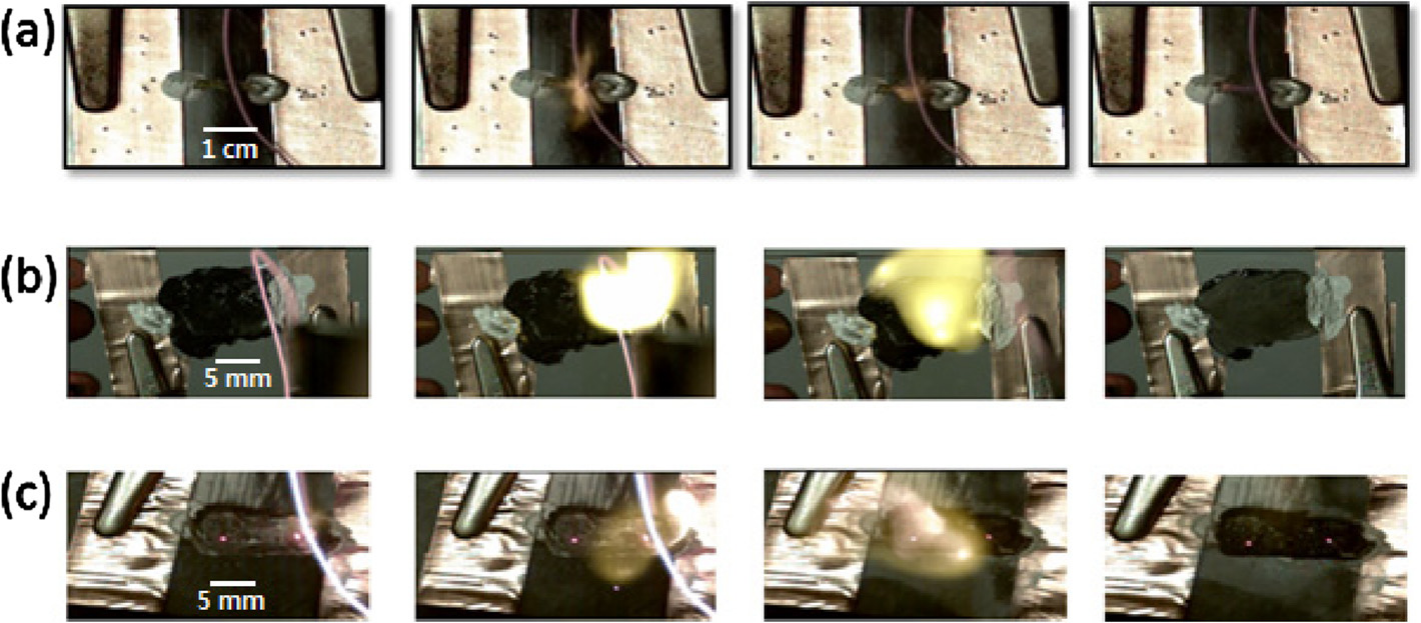
**Thermopower wave propagation in various CNT/fuel composites. (a)** 1D CNT/fuel composite (aligned array), **(b)** 2D CNT/fuel composite (film), and **(c)** 3D CNT/fuel composite (cluster).

Typical combustion events in the 1D, 2D, and 3D CNT/fuel composites are shown in Figure 4. In the 1D CNT/fuel composite arrays, the chemical reaction propagated along the axis of the array of CNTs (Figure 4a). In the 2D CNT/fuel composite film, combustion manifested as a two-dimensional diffuse reaction front, similar to the wetting of a porous film by liquid (Figure 4b). For the 3D CNT/fuel composite clusters, the combustion did not propagate in any specific direction, and the overall reaction was nearly randomly distributed in this structure (Figure 4c). An evaluation of these fundamentally different modes of combustion for these different structures provides a valuable insight into the structural effects of the core nanomaterials on the thermopower waves.

**Figure 5:**
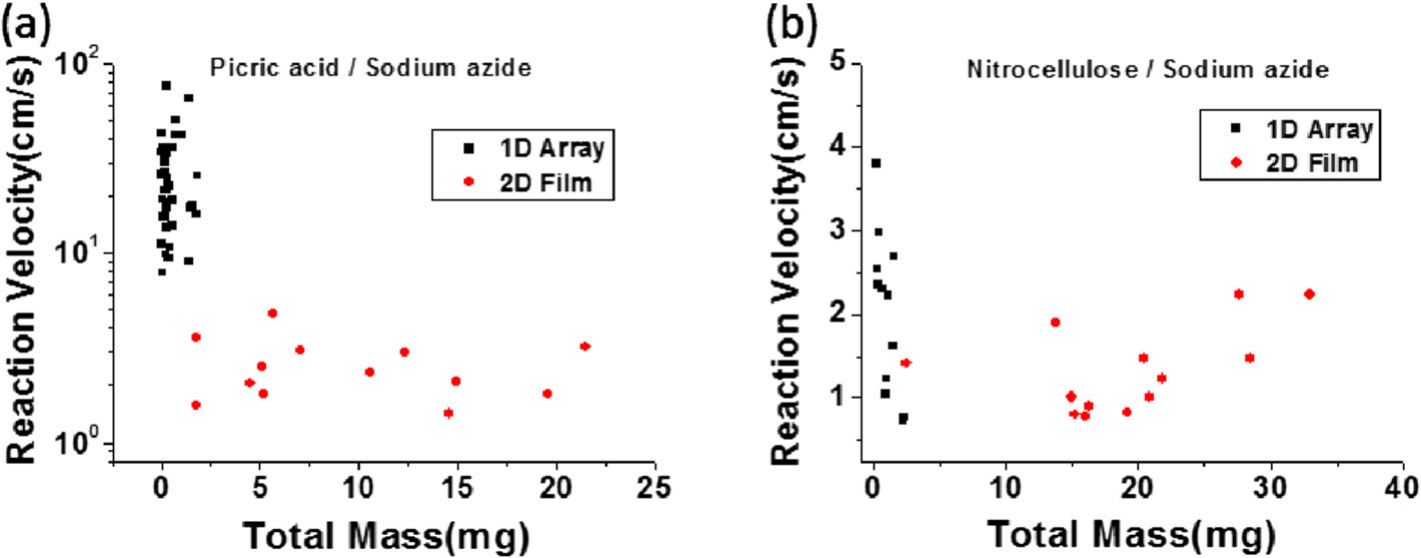
**Reaction velocity of the thermopower waves in 1D and 2D CNT/fuel composites. (a)** CNT/picric acid/sodium azide composites and **(b)** CNT/picric acid/sodium azide composites.

A comparison of reaction front velocities revealed which structure was able to transfer its reaction most efficiently. The 3D CNT/fuel composite generated essentially random combustion behavior, lacking any clear reaction front, so it was not possible to determine the velocity of the chemical reaction. The propagation of reaction had been randomly distributed inside and outside of the 3D CNT random networks. However, the 1D and 2D CNT/fuel composites revealed clear reaction fronts of the thermopower wave, and the reaction velocities were determined from the high-speed microscopic images. Especially, in the 2D CNT films, the reaction was propagated as fan shapes from the starting position on the surface of the films, similar to getting wetted, and the reaction front on the films were possible to be measured by a high-speed microscopic setup. The average reaction velocity in the 1D CNT/fuel composite was much faster than that in the 2D CNT/fuel composite, as shown in Figure 5. Moreover, the reaction velocities in specimens with different chemical fuels were significantly different. In the picric acid/sodium azide case, the reaction velocities in the 1D and 2D CNT/fuel composites were 24.5 and 2.3 cm/s, respectively, while the corresponding values were 2.0 and 1.2 cm/s in the case of nitrocellulose/sodium azide. These differences in the reaction velocities might be due to the difference in thermal diffusivity of the aligned 1D CNT array and the randomly oriented 2D CNT film. It is known that the thermal diffusivity of vertically aligned CNTs is 70 times greater in the vertical direction than in the orthogonal direction [22]. Due to this highly anisotropic heat transfer property of the 1D CNT array [23,24], the majority of the energy released from the chemical reaction was effectively transferred in the longitudinal direction, and this resulted in the faster reaction velocity compared to that in the 2D CNT film.

**Figure 6:**
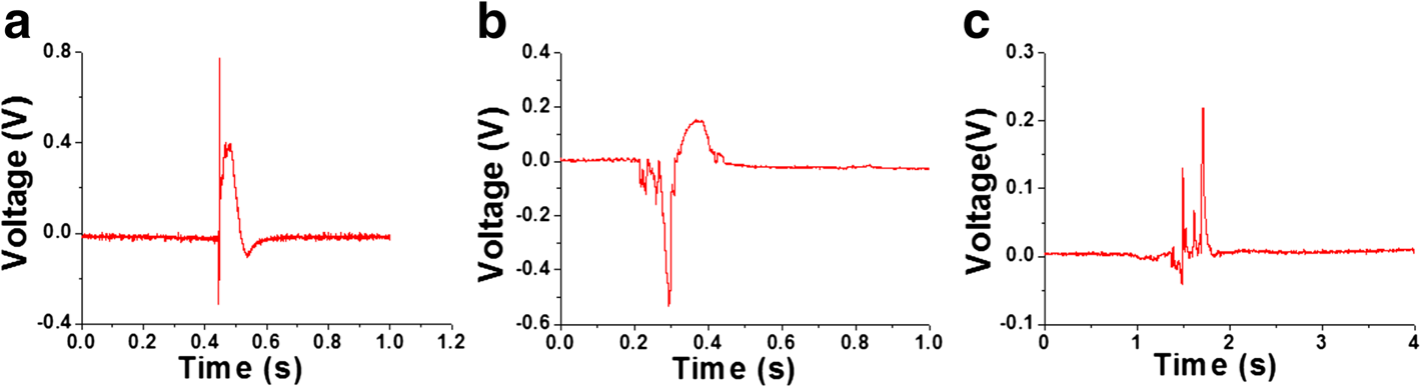
**Typical voltage signals from thermopower waves. (a)** 1D, **(b)** 2D, and **(c)** 3D CNT/fuel composites.

**Figure 7:**
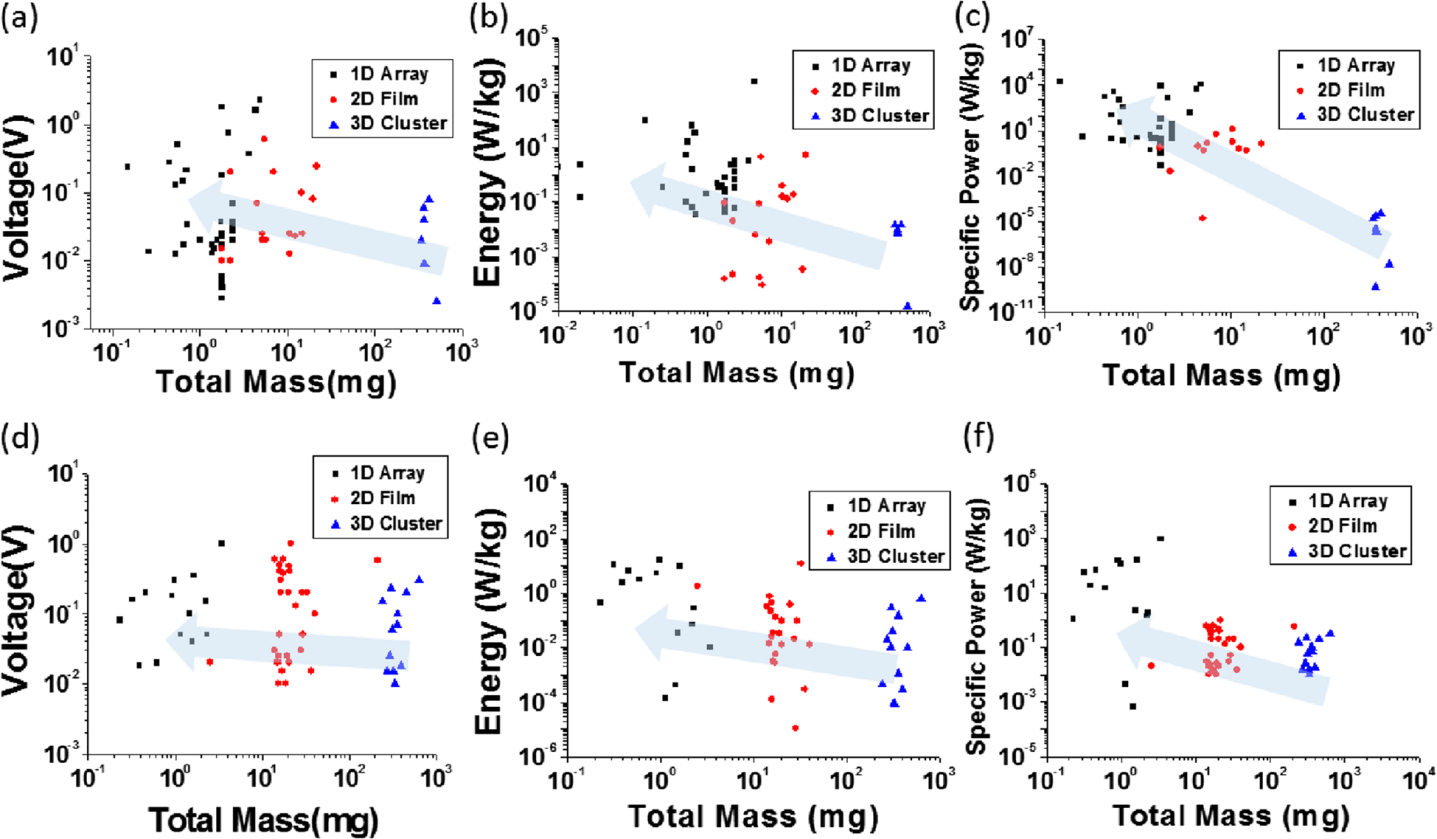
**Scatter plots of energy generation from thermopower waves. (a)** Peak voltage, **(b)** energy generation, and **(c)** specific power from thermopower waves for the CNT/picric acid/sodium azide composites. Scatter plots of **(d)** peak voltage, **(e)** energy generation, and **(f)** specific power for the CNT/nitrocellulose/sodium azide composites.

The different combustion modes depending on the particular structures produced diverse voltage signals that were synchronized with the thermal distributions from the thermopower waves, as shown in Figure 6. First, no matter what the type of fuel and the CNT structure, the characteristic fluctuation of the voltage signal associated with thermopower waves was observed, similar to the findings of previous work [10]. After this initial behavior subsided, the well-controlled thermopower waves in the 1D CNT/fuel composite typically generated a single-polarity voltage signal induced by a direction-temperature gradient in the aligned CNTs during combustion (Figure 6a). On the other hand, thermopower waves in the 2D CNT/fuel composite generally produced a double-polarity voltage signal that was produced by either the inversion of the temperature gradients or the thermal wave propagation in multiple directions on the 2D CNT film (Figure 6b). In the 3D CNT/fuel composite, the voltage signal from the thermopower waves possessed a more chaotic behavior due to the randomly propagating thermal waves, as described in the discussion about reaction velocities above (Figure 6c).To evaluate the energy generated from the thermopower waves, the peak voltages, the energy generation per unit mass, and the specific power were evaluated in the CNT/fuel composites; these data are shown in Figure 7. The average peak voltages of the 1D and 2D CNT/fuel composites were similar (175 and 165 mV, respectively), while the absolute magnitude of the voltage in the 3D CNT/fuel composite was relatively small (82 mV) (Figure 7a). However, the energy generation per unit mass, calculated by integrating the voltage signal, indicated different characteristics in the different structures (Figure 7b). Energy generation increased in the following order: 1D, 2D, and 3D CNT structures. This means that the overall energy generation per unit mass is highest in the one-dimensional aligned structure, while random orientations of CNTs show diminished energy generation. Another significant factor is the specific power that determines the maximum transient energy obtained from the thermopower waves. As with the energy generation per unit mass, the 1D CNT/fuel composites produced the largest specific power, while the 3D CNT/fuel composite provided the least (Figure 7c). To independently confirm the structural effects, these three characteristics of the thermopower waves were measured using a different fuel mixture, nitrocellulose/sodium azide (Figure 7d,e,f). The overall aspects of energy generation were exactly the same as those with the picric acid/sodium azide case, except the absolute magnitudes of each. This suggests that the structural effects resulting from the different alignments would be common to composites with an assortment of fuel layers.

**Figure 8:**
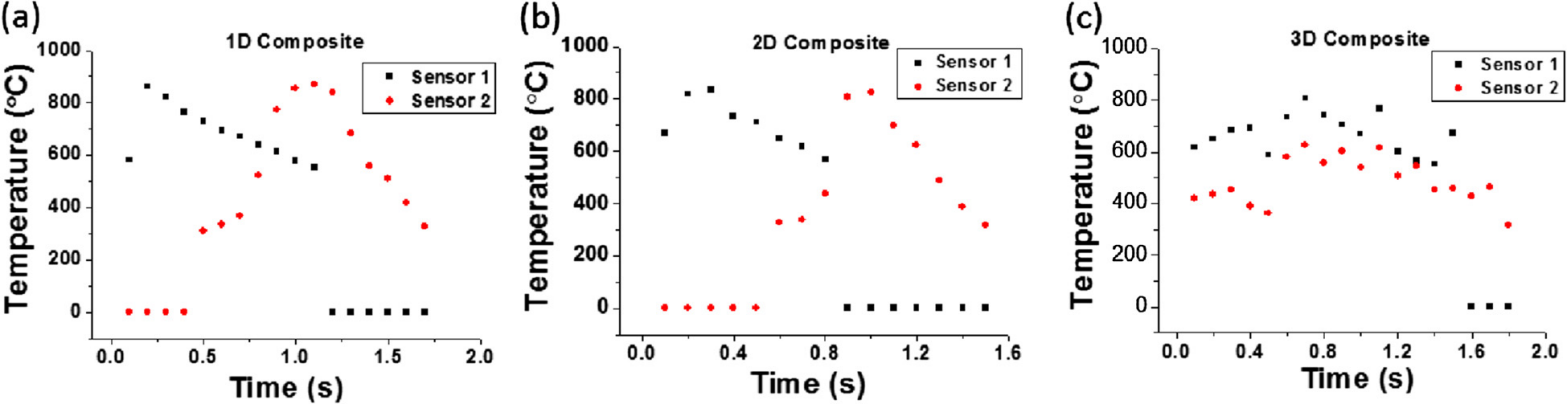
**Temperature distribution of CNTs in thermopower waves. (a)** 1D, **(b)** 2D, and **(c)** 3D CNT/fuel composites.

The marked difference in the absolute magnitude in energy generation between the picric acid/sodium azide and the nitrocellulose/sodium azide cases was due to the reconstruction of the chemical structures in fuel mixtures. Figure 3a clearly shows the one-dimensional cylindrical shape of the picric acid/sodium azide coatings, while the nitrocellulose/sodium azide mixtures are randomly dispersed between the CNT nanostructures (Figure 3d). The picric acid/sodium azide mixtures formed 2,4,6-trinitro sodium phenoxide ((NO_2_)_3_C_6_H_2_ONa) and hydrogen azide (H-N3) in DI water when the H^+^ and Na^+^ ions are exchanged because the picric acid is more acidic than hydrazoic acid (HN_3_). In this condition, the π-stacked benzene structure can be constructed by replacing the hydroxide group, which induces the hydrodynamic alignment of fuel mixtures. The polarity difference between picric acid (2,4,6-trinitro phenol) and the 2,4,6-trinitro sodium phenoxide induces continuous benzene stacking, which leads to the formation of the one-dimensional cylindrical structures of the fuel mixtures, like the unique fuel alignment in Figure 3a. This continuous, aligned structure of the fuel mixtures can enhance combustion in the thermopower waves, as shown in the comparison of Figure 5a,b, and this results in amplified energy generation in the picric acid/sodium azide mixtures. In the nitrocellulose/sodium azide with CNT network, the energy transfer is relatively slow due to lots of grain boundaries among the chemical fuels. On the contrary, in the picric acid/sodium azide with CNT network, by means of the formation of one-dimensional cylindrical structure of the chemical fuels, the energy transfer and the combustion rate are quite enhanced and amplified due to the relative decrease of the boundaries for the propagation in the same dimension. Moreover, the new chemicals, 2,4,6-trinitro sodium phenoxide and hydrogen azide (H-N3) in the picric acid/sodium azide, have the negative enthalpy of formation, and they enhance the overall energy generation as well.The real-time temperature distribution, as measured by the two optical pyrometers, can elucidate how the alignment of the CNTs affects the shape of voltage signal, as well as the overall energy generation resulting from the thermopower waves. The two optical pyrometers were placed at both ends of the CNT structures to record the temperature distributions and changes as the thermopower wave passed, as shown in Figure 8. In general, at the beginning of the reaction, the temperature at the ignition position should be higher than that at the opposite end. After the reaction propagated through the core materials, the temperature at the opposite side should be at its maximum value due to the position of the reaction front. In the 1D CNT array (Figure 8a), the temperature at the ignition position quickly increased up to 870°C, whereas the temperature at the opposite side was constant. While the thermopower waves propagated through the 1D aligned CNT array, the temperature at the ignition point cooled in the ambient surroundings. At the same time, the temperature at the opposite side gradually increased owing to the heat transfer through the aligned CNTs. When the thermopower waves finally reached the end of the CNT structures, the temperature at the opposite side was maximum, about 870°C, and the temperature at the ignition position was around 590°C. This data indicated that the temperature at the ignition position was still high, although the reaction front of the thermopower waves had reached the end position. Therefore, the voltage signal in the 1D CNT/fuel composite did not possess a clear polarity inversion, as shown in Figure 6a. In the 2D CNT film structures (Figure 8b), when the reaction started with ignition, the overall temperature gradient was similar to that of the 1D CNT structures. However, before the thermopower waves reached the ending position, the temperature at the ignition point decreased below the minimum measurable temperature of the optical pyrometer. When the propagation of the thermopower waves finished, the temperature at the ending position was close to 870°C, while the temperature at the ignition position was quite low so that the temperature gradient was largely inverted in comparison with the initial stage. Although the overall shape of the temperature gradient was similar to that of the 1D CNT/fuel composite, the inversion of the temperature gradient was sharper than that in the 1D CNT/fuel composite case. This inversion of temperature gradient caused the double polarity of the voltage signal (Figure 6b) that distinguished the energy generation from thermopower waves in the 2D and 1D CNT/fuel composites. The 3D CNT/fuel composite exhibited totally different temperature gradients (Figure 8c). When the 3D CNT clusters were fabricated, they were randomly aggregated and the interior network was not uniform. Thus, the heat would be conducted randomly along this network. When the thermopower waves were launched, the thermal waves propagated randomly in all directions, and this resulted in transient temperature distributions without any clear gradients. Moreover, the overall temperature distributions varied significantly across multiple experiments, and the voltage signals possessed more chaotic behaviors than did those of the 1D and 2D CNTs structures. This demonstrates that there is a correlation between the voltage signal and temperature distributions generated by thermopower waves.

The temperature distributions in the 1D, 2D, and 3D CNT structures also support the finding that the total energy generation and specific power were maximized in the 1D CNT/fuel composite. To amplify the energy generation, the charge carrier movement should proceed in one direction without being cancelled by motion in the opposite direction. As shown in Figure 6, the voltage signal from 1D CNT structures only maintained its single polarity by nearly one direction of temperature gradient (Figure 8a). However, in the 2D case, the voltage signal generally possessed double-polarity qualities induced by the inversion of the temperature gradient (Figure 8b). Moreover, thermopower waves, initiated by the ignition, spread out in multiple directions in the 2D CNT film, and this resulted in a decrease in overall energy generation and specific power (Figure 7b,c) [8]. This explanation can be applied to the 3D CNT/fuel composite also. Because the propagation was more anisotropic, which caused the mixing and inversion of the temperature gradients, the 3D CNT composite would not exhibit effective energy transfer because of cancellation among the randomly moving charge carriers. For this reason, the energy generation and specific power would be the smallest in these structures, among the three cases. Therefore, it is demonstrated that controlling the alignment of the core materials can modulate the overall aspects of combustion via the heat conduction along the core nanostructures.

## Conclusions

In summary, we explored the effect of the structure of large-area CNT/fuel composites on the energy generation from thermopower waves. The different types of CNT structures - 1D CNT arrays, 2D CNT films, and 3D CNT clusters - were fabricated as the core of the CNT/fuel composites. The thermopower waves in the three different nanostructured composites were characterized by the propagation of the combustion front, the real-time voltage signal, the total energy generation, and the specific power. The 1D CNT/fuel composite produced a voltage signal with a single polarity, which was driven by the unidirectional temperature gradient, and this physical characteristic resulted in relatively large energy generation per unit mass and specific power. The voltage signal of the 2D CNT/fuel composite showed a dynamic change in polarity during the propagation of the thermopower waves. The inversion of the temperature gradients between the starting time and the ending time caused this polarity change, and the change was associated with the cancellation of some of the effective charge carrier movements and a loss of energy during the conversion process in comparison with the 1D CNT structures. The voltage signal in the 3D CNT/fuel composite was highly irregular, and there were heavy losses in energy conversion owing to the randomly propagating reaction throughout the CNT network. Understanding this structural effect on energy generation from thermopower waves can help tune device designs to optimize the hybrid composites of nanomaterials and fuels, specifically, tuning based on the effective alignment of the nanomaterials. More generally, we believe that this work provides clues to understanding the chemical to thermal to electrical energy conversion inside/outside nanostructured materials.

## Competing interests

The authors declare that they have no competing interests.

## Authors’ contributions

HH, TY, KL, and WC took the tasks of fabrication of carbon nanotube/fuel composites in multiple dimensions, measurements of thermopower waves, overall data collection, and draft writing. JU, HK, JH, and WK provided the dispersed carbon nanotubes in solution phase as well as carbon nanotube networks in various alignments. All authors read and approved the final manuscript.
